# Letter regarding “Association between triglyceride-glucose (TyG) index and risk of depression in middle-aged and elderly Chinese adults: Evidence from a large national cohort study”

**DOI:** 10.17305/bb.2025.12739

**Published:** 2025-06-17

**Authors:** Seshadri Reddy Varikasuvu, Subodh Kumar

**Affiliations:** 1 Department of Biochemistry, All India Institute of Medical Sciences, Deoghar, India

**Keywords:** Triglyceride-glucose index, TyG, depression, metabolic health

## Abstract

This correspondence addresses the recent study by Xu et al. examining the relationship between the triglyceride-glucose (TyG) index and depression in older Chinese adults. The study’s identification of a J-shaped association between TyG levels and depressive symptoms adds meaningful insight into the connection between metabolic health and mental well-being. However, when considered alongside other findings, including those using combined indices, such as TyG-BMI and TyG-WHtR, the results suggest that a broader, multi-dimensional approach may offer greater predictive value. Supporting studies have linked these composite measures not only to depression but also to wider metabolic and cardiovascular risks. Additionally, other reviews highlight the potential link between TyG and more severe psychiatric conditions. The letter emphasizes the need for further research, especially longitudinal and interventional studies, to clarify causal relationships and explore whether improving metabolic health can help prevent or reduce depressive symptoms. The authors encourage continued exploration of metabolic indicators not just as risk markers but as possible targets for intervention.

Dear Editor,

We read with keen interest the recent article by Xu et al. titled “Association between triglyceride-glucose (TyG) index and risk of depression in middle-aged and elderly Chinese adults,” published in *Biomolecules and Biomedicine* [1]. The authors address an important question: whether metabolic health, as reflected by the TyG index, can help predict the development of depression in later life. By using longitudinal data from the CHARLS cohort and applying flexible modeling techniques such as restricted cubic splines, the authors provide valuable insights into the evolving field of metabolic psychiatry. This study enhances our understanding of how insulin resistance and mood disorders intersect. By identifying a J-shaped association between the TyG index and depressive symptoms—particularly above a threshold of 8.49—the authors highlight an area with significant implications for screening and prevention in aging populations. However, when considered alongside other emerging research, several aspects merit further exploration.

Firstly, the findings of Hu et al. present a somewhat different perspective. Their analysis, also utilizing CHARLS data, revealed an L-shaped association between TyG-BMI and depression, suggesting that composite metabolic indices incorporating anthropometric factors may relate differently to mental health outcomes [2]. This contrast raises the possibility that the TyG index in isolation may not fully capture the underlying risk landscape, particularly in older adults, where BMI might reflect resilience as much as risk.

Further supporting this idea, Liu et al. [3] examined TyG-based metrics, such as TyG-BMI and TyG-WHtR, finding that these metrics were not only associated with depression but also significantly predicted cardio-renal-metabolic multimorbidity (CRMM). Their findings underscore the potential advantages of multi-dimensional indices for identifying individuals at the greatest risk of overlapping physical and psychological conditions.

In parallel, a recent meta-analysis by Wan and Yu [4] provided a broader synthesis of cross-sectional studies, confirming a robust association between an elevated TyG index and greater odds of depression across various populations and methodologies. While Xu et al.’s prospective design adds weight to the temporal dimension, these converging findings enhance the credibility of TyG as a clinically significant marker worthy of attention.

Additionally, the systematic review by Behnoush et al. [5] extends the conversation by highlighting associations between the TyG index and more severe psychiatric outcomes, including suicidal ideation and behavior. Although Xu et al. primarily focused on depressive symptoms, the potential of the TyG index to reflect broader mental health vulnerabilities invites further exploration.

We also appreciate that Xu et al. acknowledged certain limitations, particularly their reliance on single-time-point TyG measurements and self-reported depressive symptoms. Building on that transparency, we suggest that future studies might benefit from integrating composite indices like TyG-BMI or TyG-WHtR, examining time-varying exposures, and testing mediation and interaction effects involving psychosocial, behavioral, and anthropometric variables. These refinements could strengthen the predictive validity of metabolic indices for mental health outcomes.

Lastly, we note an important gap in the current literature. Despite consistent observational evidence, a search of PubMed reveals no published randomized controlled trials or clinical interventions exploring the modification of the TyG index and its effects on depression outcomes. This lack of experimental data limits causal interpretation and presents a clear direction for future research. Interventional studies are needed to test whether improving metabolic health through lifestyle or pharmacological means can reduce the risk or severity of depressive symptoms. The study by Xu et al. adds valuable insight to this important field. As research continues to explore the link between metabolism and mental health, we support their call for more studies. We also encourage viewing metabolic factors not just as measurements but as potential targets for preventing and treating depression.
